# Clinical Characteristics and Abnormal Parameters Evolution in Patients With Novel Coronavirus Infection: A Case Series of 272 Cases in Guangzhou

**DOI:** 10.1017/dmp.2021.149

**Published:** 2021-05-18

**Authors:** Yubing Wang, Zhongwei Hu, Jie Luo, Fuchun Zhang, Lianjiao Huang, Hao Li, Xueliang Wen, Yuejun Pan, Meihong Chen, Ruosu Ying, Huirong Jiang, Sirui Chen, Zhilin Pan, Hongkun Chen, Huimin Xu, Chunliang Lei, Yajuan Han

**Affiliations:** 1 Department of Thoracic Surgery, Guangzhou Eighth People’s Hospital, Guangzhou Medical University, Guangzhou City, P.R. China; 2 Department of Gastroenterology, Guangzhou Eighth People’s Hospital, Guangzhou Medical University, Guangzhou City, P.R. China; 3 Department of Endocrinology and Metabolism, Guangzhou Eighth People’s Hospital, Guangzhou Medical University, Guangzhou City, P.R. China; 4 Guangzhou Eighth People’s Hospital, Guangzhou Medical University, Guangzhou City, P.R. China; 5 Department of Emergency, Guangzhou Eighth People’s Hospital, Guangzhou Medical University, Guangzhou City, P.R. China; 6 Infectious Disease Center, Guangzhou Eighth People’s Hospital, Guangzhou Medical University, Guangzhou City, P.R. China

**Keywords:** COVID-19, characteristics, risk factors, severe, continuous change

## Abstract

**Objective:**

The aim of this study was to present the clinical characteristics and dynamic changes in laboratory parameters of the coronavirus disease 2019 (COVID-19) in Guangzhou, and explore the probable early warning indicators of disease progression.

**Method:**

We enrolled all the patients diagnosed with COVID-19 in the Guangzhou No. 8 People’s Hospital. The patients’ demographic and epidemiologic data were collected, including chief complaints, lab results, and imaging examination findings.

**Results:**

The characteristics of the patients in Guangzhou are different from those in Wuhan. The patients were younger in age, predominately female, and their condition was not commonly combined with other diseases. A total of 75% of patients suffered fever on admission, followed by cough occurring in 62% patients. Comparing the mild/normal and severe/critical patients, being male, of older age, combined with hypertension, abnormal blood routine test results, raised creatine kinase, glutamic oxaloacetic transaminase, lactate dehydrogenase, C-reactive protein, procalcitonin, D-dimer, fibrinogen, activated partial thromboplastin time, and positive proteinuria were early warning indicators of severe disease.

**Conclusion:**

The patients outside epidemic areas showed different characteristics from those in Wuhan. The abnormal laboratory parameters were markedly changed 4 weeks after admission, and also were different between the mild and severe patients. More evidence is needed to confirm highly specific and sensitive potential early warning indicators of severe disease.

Since early December 2019, a pneumonia of unknown origin (other than exposure history to Huanan seafood wholesale market) occurred in Wuhan, Hubei Province. It was later determined to be caused by severe acute respiratory syndrome coronavirus 2 (SARS-COV-2). On February 11, 2020, WHO named the pneumonia caused by this novel coronavirus as COVID-19 (coronavirus disease 2019). Cumulative infections in the world now exceed 146 million, with over 3 million deaths as of late April 2021.

At the end of March 2020, there were more than 80,000 confirmed cases of COVID-19 pneumonia in China, of which more than 67,000 cases were in Hubei Province, where more than 95% of the 3,000 deaths occurred. In the early stage of the rapid outbreak of this epidemic, medical resources in Hubei could not quickly respond to the treatment needs of more than 4,000 newly confirmed cases each day. They were able to provide little more than symptomatic treatment, resulting in a large proportion of infected people and deaths in the region. However, the progress of disease transmission slowed down rapidly with the understanding of transmission routes, the advent of supplemental medical resources, and the popularization of mass prevention education.

At present, SARS-COV-2 has spread worldwide, with increasing risk of infection.^1^ Most epidemic modes are not like the concentrated outbreak in Hubei Province, but are closer to what has been seen in other provinces of China,^2^ where sporadic cases recur for a long time. We believe that analyzing case characteristics that come from outside epidemic areas will help us understand the disease from another perspective. Guangdong Province is the province with the largest number of cases, barring Hubei. As the central city of the Pearl River Delta, many people converge in Guangzhou, making it a huge base for a floating population. The floating population, also known as migrant workers, was a high-risk population during this epidemic situation, because of meeting more people due to the nature of their work. It caused the difference in patient characteristics between Guangzhou and Wuhan. By March 2020, there were more than 300 confirmed cases and only 1 death in Guangzhou. These cases can serve to represent patient characteristics outside the epidemic area cities. No. 8 People’s Hospital is the appointed hospital for COVID-19 outpatients, and we received more than 80% of the patients in Guangzhou to reflect the characteristics found outside epidemic areas.

To reduce mortality, it is critically important to understand the rules of the disease and find the predictive indicators of disease severity as early as possible. This study summarized the clinical data of inpatients with COVID-19, and analyzed them according to the grouping of clinical mild/normal and severe/critical classification to explore the high-risk factors related to severe disease.

## Methods

### Patients and Diagnostic Criteria

All patients infected by the novel coronavirus admitted to Guangzhou No. 8 People’s Hospital from January 22, 2020, to February 15, 2020, were enrolled as research subjects, except minor patients less than 14 years old and pregnant women. Suspected cases were screened according to the “diagnosis and treatment protocol for novel coronavirus pneumonia (the fifth trial version)” that was published by the National Health Commission of the People’s Republic of China and the National Administration of Traditional Chinese Medicine. If the clinical symptoms were consistent with the criteria, respiratory tract secretions and other samples were acquired for real-time fluorescence reverse transcription-polymerase chain reaction (RT-PCR) to detect the presence of COVID-19, using the 2019-nCoV (ORF1ab/N) nucleic acid detection kit (Bio-germ, Shanghai, China). Patients who tested positive for the nucleic acids of this coronavirus were identified as confirmed cases and enrolled in the study. All diagnoses were confirmed by a group of experts from No. 8 People’s Hospital of Guangzhou. A total of 267 patients (98.16%) were discharged with improvement or cure, the average hospitalization day was 19.94 ± 8.10 days, and 1 patient (0.4%) died, up to March 13, 2020.

The study was approved by the ethics committee in Guangzhou No. 8 People’s Hospital (No. 202001134), and the signature of informed consent was waived due to the retrospective nature of the current study and because the data do not divulge any private information of the patients.

### Data Collection

In this current study, we collected general information from all patients, including age, gender, generational classification of infectious diseases, history of combined chronic diseases, and smoking history. Clinical symptoms were also recorded according to the chief complaint on admission and physical examination results. The generational classification of infectious diseases was defined according to the epidemiological history of COVID-19, as first generation (patients with direct exposure to the Huanan seafood wholesale market, or infected with the virus other than by other people), the second generation (those transmitted by the first generation patients, and those came from Wuhan or who have been to Wuhan recently), the third generation (those infected by the second generation patients), and the fourth generation (those infected by the third generation of infected persons all over the country).

The dynamic changes in laboratory parameters and imaging examination data on admission were gathered from medical records, including routine blood exams, myocardial enzymogram, coagulation function, D-dimer, arterial blood gas analysis, blood lactate, procalcitonin, urine routine examination, and chest computed tomography (CT) examination. All examinations were conducted by the central laboratory of the hospital and imaging department.

### Statistical Analysis

SPSS 20.0 was used for statistical analysis. The measurement data of normal distribution were shown as mean ± SD, and t-test was used for comparison between the two groups. For the data of abnormal distribution, it was shown as median (first to third quartile), and nonparametric rank sum test was used for comparison. Chi squared test or rank sum test were used to compare the counting data among groups, which was shown as exact number (%). A *P* value < 0.05 was defined as statistically significant.

We defined the patients to be mild/normal or severe/critical with/without clinical symptoms, with/without pneumonia and the severity, with/without respiratory failure, shock, or other organ failure.

## Results

A total of 272 patients were enrolled in this study. Among these patients, 126 of them were males and 146 females with a gender ratio of 0.86 (male/female). The range of age was from 15 to 90 years, and the average age was 48.7 ± 15.9 years. According to the generational classification of infectious patients, 170 (62.5%) of them were first or second generation, 44 (16.2%) were third generation, and 58 (21.3%) were fourth generation. Patients with history of smoking or current smoking was 7.7% (21/271), and there was no significant difference between the two groups (Table 1). As for the combined disease, the most common comorbidity was hypertension (17.7%; 48/272), followed by diabetes (6.6%; 18/272), and cardiovascular disease (4.4%; 12/272).

**Table 1.** Demographic and epidemiologic baseline data

	**Total** **(** ***N*** **= 272)**	Clinical classification		Statistic	***P*** **-Value**
		**Mild and normal** **(** ***N*** **= 236)**	**Severe and critical** **(** ***N*** **= 36)**		
Gender				*χ*^2^ = 5.149	0.023
Male	126(46.32%)	103(43.64%)	23(63.89%)		
Female	146(53.68%)	133(56.36%)	13(36.11%)		
Age	48.65 ± 15.88	46.84 ± 15.73	60.53 ± 11.20	*t* = 6.430	<0.001
Generation classification				*Z* = 0.944	0.345
First and second generation	170(62.50%)	144(61.02%)	26(72.22%)		
Third generation	44(16.18%)	42(17.80%)	2(5.56%)		
Fourth generation	58(21.32%)	50(21.19%)	8(22.22%)		
Smoking history or current smoking^a^				*χ*^2^ = 0.021	0.886
Yes	21(7.75%)	19(8.05%)	2(5.71%)		
No	250(92.25%)	217(91.95%)	33(94.29%)		
Comorbidities					
Hypertension	48(17.65%)	36(15.25%)	12(33.33%)	χ^2^ = 7.025	0.008
Cardiovascular disease	12(4.41%)	8(3.39%)	4(11.11%)	χ^2^ = 2.775	0.096
Diabetes	18(6.62%)	14(5.93%)	4(11.11%)	χ^2^ = 0.647	0.421
Malignant tumor	5(1.84%)	3(1.27%)	2(5.56%)		0.132
Cerebrovascular disease	7(2.57%)	6(2.54%)	1(2.78%)		1.000
COPD	2(0.74%)	0(0.00%)	2(5.56%)		0.017
Kidney disease	3(1.10%)	1(0.42%)	2(5.56%)		0.047
Thyroid disease	5(1.84%)	5(2.12%)	0(0.00%)		1.000
Chronic liver disease	7(2.57%)	6(2.54%)	1(2.78%)		1.000

a One patient in the severe and critical group missed providing the data for smoking history or current smoking.

Abbreviation: COPD, chronic obstructive pulmonary disease.

In comparing the mild/normal and severe/critical groups, there are more male patients in the severe and critical group (with a gender ratio of 1.77; *P* = 0.023). At the same time, elderly patients were more likely to progress to severe and critical situation (46.8 ± 15.7 vs 60.5 ± 11.2 in the two groups, respectively; *P* <0.001). Several comorbidities had a higher proportion of patients in the severe/critical group, such as hypertension, chronic obstructive pulmonary disease (COPD), and kidney disease.

### Clinical Symptoms

Fever was the most common symptom on admission, with 75% (204/272) of patients reporting with elevated fever. The normal reference value of 36.9 °C was used to calculate the average temperature in both groups. Although the proportion of fever was higher in the severe and critical group vs mild and normal group (72.5% vs 91.7%, respectively; *P* = 0.013; Table 2), the exact temperature was not significantly different between the two groups (Table 3). The other most common symptoms were cough (62.1%; 169/272), expectoration (25.7%; 70/272), pharyngeal discomfort (24.6%; 67/272), and shiver (20.6%; 56/272) (Table 2).

**Table 2.** Clinical symptoms of the patients

Vaiable	Total (*N* = 272)	Clinical classification		Statistic	*P*-Value
		Mild and normal (*N* = 236)	Severe and critical (*N* = 36)		
Fever	204(75.00%)	171(72.46%)	33(91.67%)	*χ*^2^ = 6.147	0.013
Cough	169(62.13%)	142(60.17%)	27(75.00%)	*χ*^2^ = 2.920	0.088
Expectoration	70(25.74%)	57(24.15%)	13(36.11%)	*χ*^2^ = 2.337	0.126
Pharyngeal discomfort	67(24.63%)	57(24.15%)	10(27.78%)	*χ*^2^ = 0.221	0.638
Shiver	56(20.59%)	43(18.22%)	13(36.11%)	*χ*^2^ = 6.115	0.013
Muscle soreness	46(16.91%)	36(15.25%)	10(27.78%)	*χ*^2^ = 3.486	0.062
Fatigue	41(15.07%)	31(13.14%)	10(27.78%)	*χ*^2^ = 5.231	0.022
Chest tightness	40(14.71%)	33(13.98%)	7(19.44%)	*χ*^2^ = 0.743	0.389
headache	28(10.29%)	24(10.17%)	4(11.11%)	*χ*^2^ = 0.000	1.000
Nasal congestion and runny nose	25(9.19%)	23(9.75%)	2(5.56%)	*χ*^2^ = 0.251	0.616
Anorexia	16(5.88%)	10(4.24%)	6(16.67%)	*χ*^2^ = 6.616	0.010
Dizzy	13(4.78%)	7(2.97%)	6(16.67%)	*χ*^2^ = 10.048	0.002
Diarrhea	9(3.31%)	7(2.97%)	2(5.56%)	*χ*^2^ = 0.095	0.757
Wheezing	1(0.37%)	1(0.42%)	0(0.00%)		1.000
Nausea	7(2.57%)	6(2.54%)	1(2.78%)		1.000
Vomit	6(2.21%)	5(2.12%)	1(2.78%)		0.577
Chest pain	4(1.47%)	2(0.85%)	2(5.56%)		0.086
Dry mouth	2(0.74%)	2(0.85%)	0(0.00%)		1.000
Bitter taste	2(0.74%)	1(0.42%)	1(2.78%)		0.248
Lumbago	2(0.74%)	1(0.42%)	1(2.78%)		0.248
Eye pain	2(0.74%)	2(0.85%)	0(0.00%)		1.000
Itching eyes	1(0.37%)	1(0.42%)	0(0.00%)		1.000
Head inflation	1(0.37%)	1(0.42%)	0(0.00%)		1.000
Dyspnea	1(0.37%)	1(0.42%)	0(0.00%)		1.000

**Table 3.** Laboratory and imaging examinations of the patients

Variable	**Total** **(** ***N*** **= 272)**	Clinical classification		Statistic	***P*** **-Value**
		**Mild and normal** **(** ***N*** **= 236)**	**Severe and critical** **(** ***N*** **= 36)**		
Temperature, median (IQR)	36.9 (36.5–37.4)	36.9 (36.5–37.4)	37.0 (36.5–38.3)	Z = 1.655	0.098
Respiratory rate, median (IQR)	20.0 (18.0–20.0)	20.0 (18.0–20.0)	20.0 (20.0–21.0)	Z = 2.799	0.005
Heart rate, median (IQR)	84.0 (78.0–93.0)	85.0 (78.0–93.0)	83.5 (77.0–95.5)	Z = 0.032	0.975
Arterial partial pressure of oxygen (mmHg), median (IQR)	93.2 (81.2–108.0)	96.0 (83.2–108.0)	73.5 (69.7–83.0)	Z = 5.003	<0.001
Leukocyte count (10^9^/L)	4.69 (3.75–5.86)	4.62 (3.73–5.81)	4.93 (4.19–6.54)	Z = 1.529	0.126
Lymphocyte absolute value(10^9^/L)	1.34 (1.00–1.88)	1.42 (1.09–1.95)	0.90 (0.55–1.10)	Z = 5.590	<0.001
PLT count (10^9^/L)	184.0 (148.0–227.0)	187.0 (148.0–230.0)	170.0 (143.5–198.0)	Z = 1.997	0.046
Creatine kinase (U/L)	72.0 (49.0–112.0)	68.0 (48.0–103.0)	123.0 (79.0–212.0)	Z = 3.888	<0.001
Glutamic oxaloacetic transaminase (U/L)	20.9 (17.2–28.6)	20.1 (16.7–26.2)	32.1 (27.0–47.4)	Z = 5.600	<0.001
Creatine kinase isoenzyme (U/L)	10.8 (8.6–14.9)	10.8 (8.8–15.3)	11.0 (8.0–14.4)	Z = 0.412	0.681
Lactate dehydrogenase(U/L)	186.0 (152.0–241.0)	179.0 (150.0–222.0)	305.5 (216.0–396.0)	Z = 5.124	<0.001
Blood lactic acid (mmol/L)	1.77 (1.4–2.1)	1.70 (1.30–2.10)	1.80 (1.45–2.20)	Z = 1.002	0.317
CRP (mg/L)	30.02 (19.8–44.5)	26.55 (18.11–36.96)	37.02 (23.92–63.66)	Z = 2.854	0.004
Procalcitonin (ng/mL)	0.04 (0.03–0.06)	0.04 (0.03–0.06)	0.09 (0.05–0.19)	Z = 5.431	<0.001
D-dimer (ug/L DDU)	1110.0 (780.0–1580.0)	1090.0 (740.0–1480.0)	1700.0 (1150.0–1950.0)	Z = 4.078	<0.001
Urinary occult blood				χ^2^ = 0.855	0.355
Negative	113(60.75%)	97(59.51%)	16(69.57%)		
Positive	73(39.25%)	66(40.49%)	7(30.43%)		
Urinary protein				χ^2^ = 14.009	<0.001
Negative	153(82.26%)	141(86.50%)	12(52.17%)		
Positive	33(17.74%)	22(13.50%)	11(47.83%)		
FbG				χ^2^ = 10.016	0.002
Normal	148(57.4%)	137(61.2%)	11(32.3%)		
Abnormal	110(42.6%)	87(38.8%)	23(67.7%)		
Value among the abnormal (g/L), (IQR)	4.77 (4.26–5.35)	4.70 (4.26–5.18)	5.35 (4.45–6.29)	Z = 2.515	0.012
APTT				χ^2^ = 9.961	0.002
Normal	202(77.1%)	183(80.3%)	19(55.9%)		
Abnormal	60(22.9%)	45(19.7%)	15(44.1%)		
Value among the abnormal(Seconds), (IQR)	45.5 (43.9–47.5)	45.8 (44.2–47.8)	44.7 (42.5–46.9)	Z = 1.246	0.213

Abbreviation: PLT, platelet; DDU, D-dimer unit; CRP, C-reactive protein; FbG, fibrinogen; APTT, activated partial thromboplastin time.

### Physical, Laboratory, and Imaging Examinations on Admission

Upon admission, the average arterial partial pressure of oxygen is normal, at 93.2 mmHg. There are 18.0%, 32.7%, and 11.8% of patients with decreased leukocyte count, lymphocyte (LYM) absolute value, and platelet (PLT) count, respectively. In urine routine examination, 39.9% of the patients had urinary occult blood, and 17.7% had urinary protein. As for coagulation function, fibrinogen (FbG) was raised in 42.6% of patients, and activated partial thromboplastin time (APTT) was raised in 22.9%.

The dynamic changes in some lab parameters were tracked from day 1 to 4 weeks until discharge (Table 4). Compared with baseline, the level of white blood count (WBC), LYM, and PLT shown the same trends, significantly back higher in 1 and 2 weeks and still at a high level in the following weeks. There are 9.93% of patients with increased alanine aminotransferase (ALT) and without any significant different in 4 weeks. Aspartate transaminase (AST) was elevated in 14.76% of patients at baseline and reduced in 1-4 weeks. Total bilirubin (TBIL) and direct bilirubin (DBIL) were decreased at 2-3 weeks, while the blood urea nitrogen (BUN) and creatinine (Cr) were increased significant at 1-2 weeks. A total of 42.64% and 53.79% of the patients showed an increase in FbG and D-dimer at baseline, respectively. The evolution process showed that FbG and D-dimer were increased significantly in the first week after admission, and continued to 2 weeks. After 2 weeks, FbG and D-dimer were decreased to the baseline level. A total of 42.6% of patients showed procalcitonin elevation on admission; levels were still elevated at 4 weeks but without significant difference.

**Table 4.** Laboratory examinations of the patients in 4 weeks

Variable	Week 0	Week 1	Week 2	Week 3	Week 4
WBC (3.5-9.5 10^9^/L)	4.73 (3.75,5.83)	5.46* (4.41,6.80)	5.65* (4.64,6.83)	5.28* (4.42,6.73)	5.46 (4.66,6.46)
LYM (1.1-3.2 10^9^/L)	1.35 (0.99,1.89)	1.51* (1.13,2.03)	1.67* (1.33,2.06)	1.59* (1.31,1.85)	1.40* (1.19,1.82)
PLT (125-350 10^9^/L)	183.00 (144.00, 227.00)	233.00* (183.00, 285.50)	245.00* (199.00, 294.00)	203.00* (167.00, 241.00)	214.00* (153.50, 240.00)
AST (15-40 U/L)	20.90 (16.85, 29.40)	18.20* (14.75, 24.55)	16.90* (13.68, 22.65)	17.30* (13.35, 21.96)	19.20 (13.43, 30.38)
ALT (9-50 U/L)	21.50 (14.10, 33.03)	22.55 (14.78, 35.45)	24.00 (15.70, 35.70)	22.55 (14.86, 38.66)	22.80 (16.96, 50.30)
TBIL (0-26 umol/L)	9.45 (6.44, 13.41)	9.91 (7.02, 13.58)	8.86 (6.36, 11.78)	8.08* (5.53, 9.16)	7.77 (5.38, 9.90)
DBIL (0-8 umol/L)	4.00 (2.80, 5.42)	4.18 (2.89, 5.70)	3.61* (2.61, 4.82)	3.39* (2.63, 4.38)	3.71 (2.38, 4.56)
BUN (3.1-9.5mmol/L)	3.85 (3.19, 4.60)	4.17* (3.23, 5.29)	4.06 (3.26, 4.86)	4.03 (3.38, 4.81)	4.25 (3.57, 5.01)
Cr (57-111 umol/L)	61.80 (50.93, 77.20)	66.15*(54.96, 80.83)	65.25* (54.50, 79.58)	67.55 (56.18, 77.70)	64.90 (56.70, 72.20)
D-dimer (<1000 ug/L DDU)	1070.00 (510.00, 1570.00)	1260.00* (790.00, 1765.00)	1490.00*(790.00,2725.00)	1590.00(845.00,2500.00)	1440.00(1065.00,2620.00)
Procalcitonin (ng/mL)	0.05(0.04-0.10)	0.04* (0.03-0.07)	0.04 (0.03-0.09)	0.08 (0.04-0.52)	0.47 (0.04-1.27)
CRP (<10 mg/L)	20.31 (10.00, 36.88)	12.19* (10.00, 25.15)	10.00* (2.23, 12.21)	#	#
FbG(2.0-4.0 g/L)	4.00 (2.93,4.62)	4.32* (3.35,5.99)	4.47* (3.19, 5.65)	3.38 (2.78, 4.39)	3.59 (2.84, 4.33)

Abbreviations: WBC, white blood count; LYM, lymphocyte; PLT, platelet; AST, aspartate transaminase; ALT, alanine aminotransferase; TBIL, total bilirubin; DBIL, direct bilirubin; BUN, blood urea nitrogen; Cr, creatinine; DDU, D-dimer unit; CRP, C-reactive protein; FbG, fibrinogen.

* *P* < 0.05 compared with baseline.

As for the mild/normal and severe/critical groups, arterial partial pressure of oxygen was significantly different; this is one of the lab examinations involved in severity grouping (median 96.0 vs 73.5; *P* < 0.001). Abnormal results were seen more in the severe and critical group, including LYM absolute value, PLT count, creatine kinase, glutamic oxaloacetic transaminase, lactate dehydrogenase, C-reactive protein (CRP), procalcitonin, D-dimer, and urinary protein. According to the subsequent analysis, APTT was higher in the severe and critical group. However, the value of FbG among the abnormal patients could reflect the risk of progressing to severe and critical (*P* = 0.012), while that of APTT did not come to a positive result (*P* = 0.213).

As for imaging examinations, all patients in the severe and critical group had the characteristic of pneumonia in both lungs according to imaging results, while the proportion of involving both lungs in mild and normal patients was 84.7% (200/236; *P* = 0.027). In the other patients, 6 involved the left lung and 30 involved the right lung alone.

## Discussion

Coronaviruses are a family of single strand positive RNA virus with envelope. Coronaviruses can be divided into α, β, γ, and δ genera, among which α and β are more likely to infect mammals.^2^ The homology of nucleotide to human SARS virus was 78%, and approximately 50% to the Middle East respiratory syndrome (MERS) virus. Compared with the SARS outbreak of 2002-2003, many more people were infected by SARS-COV-2, and the number is still increasing. But the mortality is relatively lower at 3.46%,^3^ compared with more than 10% mortality of SARS infection.^4^ Furthermore, when talking about patients outside the epidemic areas, the mortality is even lower. But for patients with comorbidities and severe symptoms, it is still important to pay close attention to deteriorating symptoms that may lead to death. With limited medical resources, it is especially important to identify severe or critical patients as early as possible, and the current study attempts to find some characteristics related to severe and critical cases.

### Epidemiologic Features

In this study, the mean age was 48 years old, which was younger than the 425 cases reported by China CDC and Hubei Province CDC,^5^ where the mean age was approximately 59 years old. The difference might be related to the age composition of the floating population in Guangzhou; most infected patients in Wuhan were community residents. The age composition in other countries might be closer to that in our study. An 18-case series from Singapore showed a median age of 47 years.^6^

According to the very first 41-case report published by Huang,^7^ there were more male patients than female. In the report from China CDC and Hubei Province CDC, 56% of patients were male. The male majority finding was similar to that seen in SARS or MERS. The mechanism that more males were affected might be related to more cells expressing angiotensin converting enzyme 2 (ACE2) gene in men than that in women, which was reported 1.66% and 0.44%, respectively. COVID-19 could infect human respiratory epithelial cells through S-protein and ACE2 interaction.^8^ Another reason for fewer female infections could be related to a more powerful innate and adaptive immunity influenced by X chromosome and gonadal hormones. But in the current study, more female patients were enrolled, while the proportion of severe/critical patients was still male-dominated, and was aligned with the opinion from the expert group on novel coronavirus prevention. The group indicated that the general gender ratio could be either male or female dominated in different regions, so gender could not be taken as a risk factor during practice. Furthermore, the results still showed an inversion result that, while more females were affected, males were at higher risk for severe and critical symptoms, which was also mentioned by the expert group.

It is of interest to note that all the smoking patients were male. The rate of smoking in mild/normal and severe/critical groups was close, but due to the small sample size at this subgroup, we did not conduct further analysis.

### Correlation Between Epidemiological Characteristics and Severity

There was a positive correlation between age and comorbidities. Fewer comorbidities was related to low average age of patients. Three patients with COPD and kidney disease achieved statistical difference, but the number of patients with COPD and kidney disease was relatively few. The proportion of hypertension in the severe/critical group was high, but still did not reach the prevalence of approximately 40% in the elderly population according to epidemiological data.^9^ In our study, comorbidities, such as cardiovascular disease or diabetes, did not reach a *P* value less than 0.05 due to the relatively small sample size in the severe/critical group. However, the comorbidity effect has been reported in some previous studies,^10^ and needs further confirmation by assembling more cases from outside epidemic areas.

As for the generation analysis, we found a trend of more first- and second-generation patients in the severe and critical group (72.2% vs 61.0%), but it did not reach a significant difference (*P* = 0.345). We suppose there is a relationship between generation and disease severity, as the severity may be related to viral load. In 40 cases associated with a department store exposure, all of them were first and second generation, and the proportion of severe was 32.5% (13/40),^11^ which was higher than the proportion of severe cases in our current study (15.3%, 26/170). Severity might possibly be related to viral load, as exposure in that report was longer than in ours (three employees were still on duty after symptom onset).

### Lab Examination

In this study, all patients had routine blood examination. Upon admission, while most patients had normal results, 18.0%, 32.7%, and 11.8% of the patients had lower leukocyte count, LYM count, and PLT count, respectively. The decreasing trend of blood cell count was the same as previous studies. Furthermore, WBC, LYM, and PLT increased significantly during the 4 weeks. The occurrence of this phenomenon may be the natural evolution of the disease, or it may be related to the use of clinical drugs, especially glucocorticoids.

In the diagnosis and treatment protocol (using the recent seventh edition), lymphocyte decline is mentioned as a common abnormality and also as an indicator of disease severity. Here, we found nearly one-third of the patients showed decreased lymphocytes, but it could be back to normal in 1-4 weeks. The difference of lymphocyte absolute value at baseline between the two groups was also significant; the median in the mild/normal group is 1.42, while it as low as 0.90 in the severe/critical group. These results were consistent with that of the protocol, and also pointed out that some patients might progress to severe quickly after admission and show a much lower result in the first routine blood examination. The reduction of PLT numbers was reported to be a result of myelosuppression at the early stage of virus infection. When comparing the two groups, more patients had abnormal blood routine examination results in the severe/critical group, and PLT number decline can be a predictor for disease severity.

At baseline examination of liver function, the abnormal increase of ALT was 9.93%, increase of AST was 14.76%, which was significantly lower than the descriptive study at hospital in Wuhan (Chen et al. 2020^12^). In the study by Chen et al., with 99 confirmed cases of COVID-19 in Wuhan, the ALT and AST levels were increased by 28% and 35%, respectively. Similarly, Zhong’s team also reported, analyzed clinical data of 1099 laboratory confirmed cases from 552 hospitals in 31 provinces or cities, the abnormal increase of ALT and AST (> 40 IU/L) was 22.2% and 21.3%, respectively.^13^ These results suggested that the proportion of liver function damage in nonepidemic areas was lower than that in epidemic areas. TBIL and DBIL were decreased significantly in 2-3 weeks and they were related with drug induced liver injury, but could be recovered after plasma exchange. Taken together, this study did not show very clear effect on liver function. SARS-COV and MERS COV infection all have a certain influence on coagulation function.^14,15^ In this study, FbG and D-dimer levels were increased significantly in the 1 week after admission, continued to 2 weeks, and decreased to the baseline level at 3-4 weeks. Moreover, it showed a significant differenc between the mild and severe patients, which suggested that there might have a relationship between coagulation dysfunction and disease severity.

Patients also showed increases in some inflammatory marker at admission, such as CRP and procalcitonin elevation. The high proportion of procalcitonin elevation at the early stage of disease might be an indicator of the unique character of COVID-19, although the mechanism is still unclear. According to a case series of 20 pediatric patients, 80% of them showed procalcitonin elevation, which was even higher than that in adults.^16^ The difference between adult and pediatric patients might help us understand the reason for procalcitonin elevation in COVID-19 in further studies. In total, inflammation and coagulation activate each other and jointly promote the progression of the disease.

Among the 185 patients tested the urine routine, 50.3% showed abnormal results. This was consistent with the results of previous studies, which showed increased proteinuria in 63% patients and serum creatinine raising in 19%, and BUN raising in 27%. The abnormality occurred in early stage disease and all of the deaths showed a moderate or higher level of renal failure.^17^ These data indicated that COVID-19 had a high risk to cause kidney injury.^18,19^ The abnormal urine indexes (occult blood or proteinuria positive) was 50.27%, suggesting nearly half of the patients had renal damage. While, BUN and Cr were increased significantly within 1-2 weeks after admission suggesting that SARS-COV-2 may have a transient effect on the kidney but could recover.

### Imaging Examination

Currently, chest CT was believed to be a reliable diagnostic method with a high sensitivity.^20,21^ All the patients showed abnormal images upon CT scanning. There were 236 cases (86.76%) of bilateral lung infection, 6 cases (2.20%) of left lung infection, and 30 cases (11.03%) of right lung infection. In unilateral lung lesions, most of the cases were in the right lung. This might due to the different physiological structure of the lungs. The left main bronchus is slender and inclined, while the right bronchus is relatively thick, short, and straight, and the tracheal ridge in the left, which means the virus can more easily contact and enter the right airway. According to previous studies, the presence of bilateral ground glass opacity and consolidation on imaging, in the appropriate clinical background, should raise a suspicion about COVID-19.^22^ Although all the patients in the severe/critical group showed bilateral involvement, the proportion in all patients was only 86.8%, even 84.8% in mild/normal patients. These numbers were similar to those seen in patients from Wuhan^23^ or other provinces.^24^ In another study, an initial and follow-up CT obtained at 4.5 days and 11.6 days on average were compared and showed progression of COVID-19. This study also mentioned that there was a moderate correlation between the days from onset and the degree of opacification on CT.^25^ According to all the aforementioned evidence, a reexamination of suspected patients is extremely necessary to diagnose whether the CT showed unilateral involvement at the early stage of disease, and this reexamination step has been listed in the diagnosis and treatment protocol. The current study did not include the follow-up imaging data, and that was a limitation.

## Conclusions

For patients infected with COVID-19 outside epidemic areas, the patient characteristics will be a little different from those in Wuhan, primarily younger age, variable gender ratio, and fewer comorbidities. The disease features, including symptoms and lab examination results, were similar, but not completely the same as those of patients in Wuhan. The most common symptoms were fever and cough. Male, aged, hypertension comorbidity, abnormal routine blood results, raised creatine kinase, glutamic oxaloacetic transaminase, lactate dehydrogenase, CRP, procalcitonin, D-dimer, FbG, APTT, and positive in proteinuria can be candidates for early warning indicators of severe disease. The abnormal parameters results, especially markedly changed in inflammatory markers, are common. Further studies to confirm the predictive capability of these indicators, and take the dynamic evolution of this disease into account, are needed.
